# Supplementary material for: Colloidal and Acid Gelling Properties of Mixed Milk and Pea Protein Suspensions
Source: Foods. 2022 May 11;11(10):1383. doi: 10.3390/foods11101383 (PMC9140544; doi:10.3390/foods11101383)
Supplement: Supplementary file 1 [file foods-11-01383-s001.zip › Supplementary Material - Figures S1 and S2 - Oliveira et al 2022 - Foods.pdf]

SUPPLEMENTARY MATERIAL – FIGURE S1

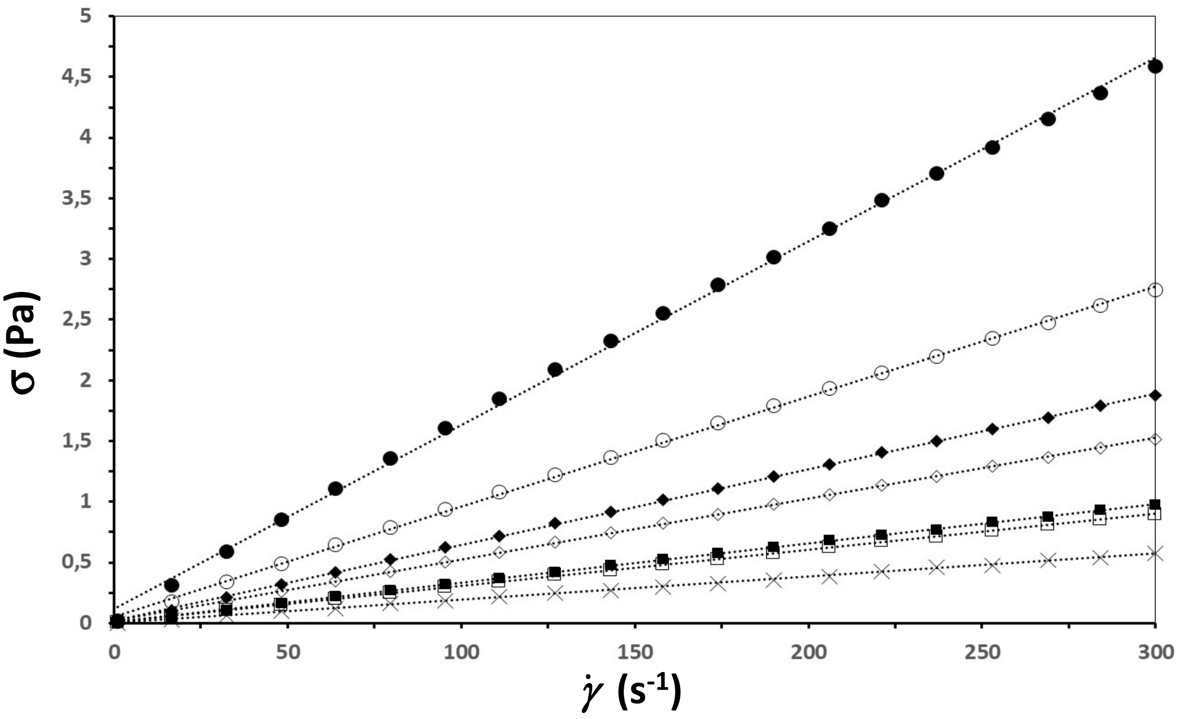

Supplementary Material 2A - Flow curves of milk (open symbols) and pea suspensions (closed symbols). The square, diamond, and circle markers represent the 5, 7 and 9% (w/w) protein, respectively. The control milk sample is represented by the “x” symbol. The Dashed Line represents the best fit Newtonian model.

SUPPLEMENTARY MATERIAL – FIGURE S2

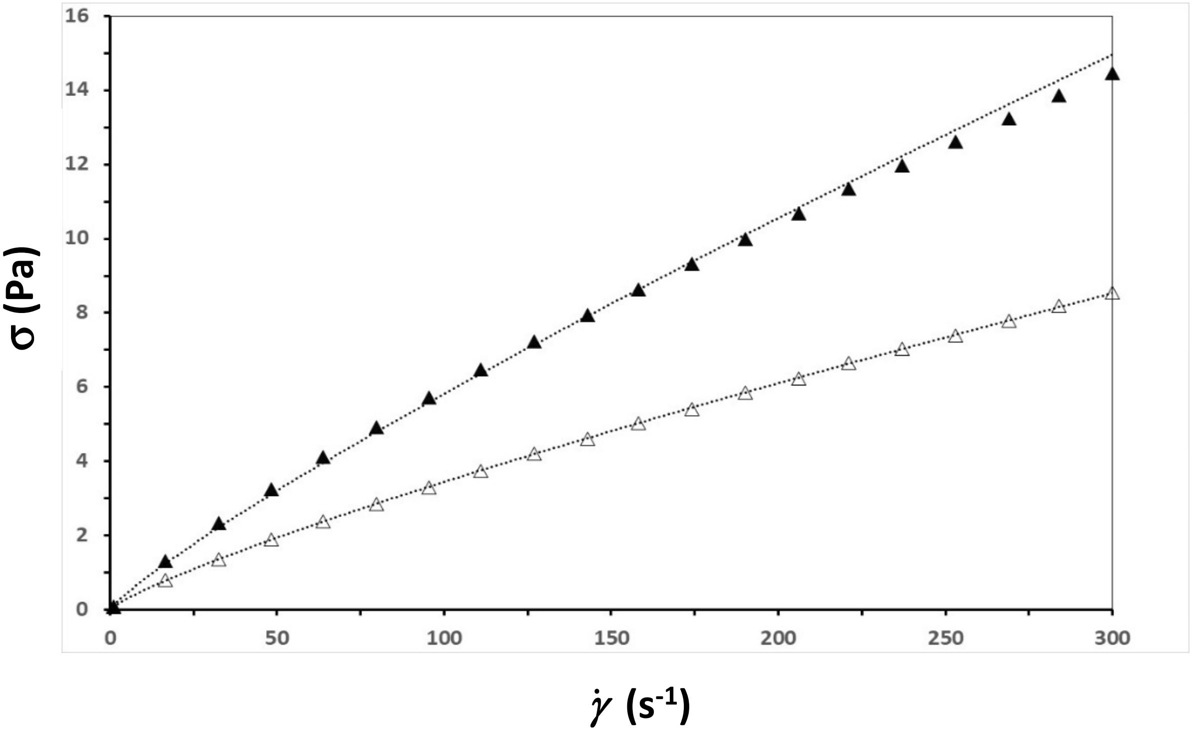

Supplementary Material 2B. Flow curves of milk (open symbols) and pea suspensions (closed symbols). The triangle marker represents the 11% (w/w) protein. The dashed line represents the best fit power-law model.
