# Supplementary material for: Colloidal and Acid Gelling Properties of Mixed Milk and Pea Protein Suspensions
Source: Foods. 2022 May 11;11(10):1383. doi: 10.3390/foods11101383 (PMC9140544; doi:10.3390/foods11101383)
Supplement: Supplementary file 1 [file foods-11-01383-s001.zip › Supplementary Material - Table S1 - Oliveira et al 2022 - Foods.pdf]

SUPPLEMENTARY MATERIAL – TABLE S1

Supplementary Material 01 - Milk responses (y) in relation to the protein concentrations (x) for skim milk and mixed milk systems. Table shows the fitted models, adjusted coefficients of determination (R^2_{adj}), regression mean squared errors ($\hat{\sigma}^2$), and F-tests for the lack of fit.

Variables	Selected Models	Lack of Fit		
		F	P	Signif.
pH				
Milk	$y = 6.9357 - 0.0830x + 0.0027x^2$	0.066	0.937	(ns)
	$R^2_{ajd} = 0.99, \hat{\sigma}^2 = 0.000270$			
Mixed	$y = 6.6084 + 0.0427x - 0.0030x^2$	0.046	0.956	(ns)
	$R^2_{ajd} = 0.59 , \hat{\sigma}^2 = 0.00028$			
Viscosity[a] (mPa.s)				
Milk	$y = 0.9447 - 0.0798x + 0.0010x^2$	1.269	0.323	(ns)
	$R^2_{ajd} = 0.99, \hat{\sigma}^2 = 0.00018$			
Mixed	$y = 1.0388 - 0.1166x + 0.0031x^2$	0.103	0.903	(ns)
	$R^2_{ajd} = 0.99, \hat{\sigma}^2 = 0.00021$			
Color (L*)				
Milk	$y = 51.0587 + 2.6517x - 0.1450x^2$	3.432	0.073	(ns)
	$R^2_{ajd} = 0.86, \hat{\sigma}^2 = 0.67100$			
Mixed	$y = 64.0835 - 2.6578x + 0.1460x^2$	0.799	0.477	(ns)
	$R^2_{ajd} = 0.77, \hat{\sigma}^2 = 1.19400$			
Color (a*)				
Milk	$y = -1.4353 - 0.8285x + 0.0518x^2$	0.981	0.408	(ns)
	$R^2_{ajd} = 0.57, \hat{\sigma}^2 = 0.15252$			
Mixed	$y = -6.4810 + 1.1083x - 0.0360x^2$	0.311	0.739	(ns)
	$R^2_{ajd} = 0.99, \hat{\sigma}^2 = 0.04300$			
Color (b*)				
Milk	$y = -10.3519 + 1.6347x - 0.0568x^2$	2.580	0.125	(ns)
	$R^2_{ajd} = 0.93, \hat{\sigma}^2 = 0.48900$			

Mixed	$y = -15.9802 + 3.8895x - 0.1766x^2$	9.656	0.005	**
	$R^2_{ajd} = 0.98, \hat{\sigma}^2 = 0.30000$			
Sed (%, m/m)				
Milk	$y = 7.0005 - 1.3858x + 0.1503x^2$	3.020	0.094	(ns)
	$R^2_{ajd} = 0.81, \hat{\sigma}^2 = 1.24500$			
Mixed	$y = -10.6600 + 4.7217x$	5.616	0.016	*
	$R^2_{ajd} = 0.98, \hat{\sigma}^2 = 3.82000$			
Ethanol (v/v)				
Milk	$y = 100.1250 - 3.2083x$	5.792	0.015	*
	$R^2_{ajd} = 0.96, \hat{\sigma}^2 = 3.51000$			
Mixed	$y = 89.8333 + 1.9167x - 0.4167x^2$	14.500	0.001	**
	$R^2_{ajd} = 0.98, \hat{\sigma}^2 = 2.71000$			

[a]Variable transformed according to the Box-Cox method ($y = viscosity^{-0.5}$). Signif. Codes: * P<0.05; ** P<0.01; ns = non-significant at 5% level.
